# ConcrEITS: An Electrical Impedance Interrogator for Concrete Damage Detection Using Self-Sensing Repairs

**DOI:** 10.3390/s21217081

**Published:** 2021-10-26

**Authors:** Jack McAlorum, Marcus Perry, Andrew C. Ward, Christos Vlachakis

**Affiliations:** Civil and Environmental Engineering, University of Strathclyde, Glasgow G1 1XJ, UK; m.perry@strath.ac.uk (M.P.); andrew.c.ward@strath.ac.uk (A.C.W.); christos.vlachakis@strath.ac.uk (C.V.)

**Keywords:** damage, concrete, repair, sensor, alkali-activated material, low-cost tomographic impedance interrogator, crack detection and location

## Abstract

Concrete infrastructure requires continuous monitoring to ensure any new damage or repair failures are detected promptly. A cost-effective combination of monitoring and maintenance would be highly beneficial in the rehabilitation of existing infrastructure. Alkali-activated materials have been used as concrete repairs and as sensing elements for temperature, moisture, and chlorides. However, damage detection using self-sensing repairs has yet to be demonstrated, and commercial interrogation solutions are expensive. Here, we present the design of a low-cost tomographic impedance interrogator, denoted the “ConcrEITS”, capable of crack detection and location in concrete using conductive repair patches. Results show that for pure material blocks ConcrEITS is capable of measuring 4-probe impedance with a root mean square error of ±5.4% when compared to a commercially available device. For tomographic measurements, ConcrEITS is able to detect and locate cracks in patches adhered to small concrete beam samples undergoing 4-point bending. In all six samples tested, crack locations were clearly identified by the contour images gained from tomographic reconstruction. Overall, this system shows promise as a cost-effective combined solution for monitoring and maintenance of concrete infrastructure. We believe further up-scaled testing should follow this research before implementing the technology in a field trial.

## 1. Introduction

Concrete degradation is an ever-growing global challenge as more structures reach the end of their design life [1]. Asset managers must detect, locate, and quantify this damage, ideally both before and after remediation. Continuous non-destructive monitoring systems for inferring and locating damage include accelerometers [2], fibre-optic sensors [3], wave propagation [4], and robotic imaging [5]. A key limitation of these techniques is that they cannot perform direct damage measurements that are both continuous in time and space (i.e., fully distributed). Other deterioration measures include field inspections to predict corrosion caused by carbonation and chloride [6]. These methods are usually destructive in nature, requiring coring of the structure [7]. Direct electrical impedance tomography and spectroscopy (EITS) measurements of the concrete itself, or of self-sensing coatings is compatible with continuous and distributed monitoring and can be non-destructive. There is precedent for EIT’s use in structural health monitoring more generally (beyond concrete), and an excellent review is provided in [8]. In short, EIT provides the ability to reconstruct a contour image of a cell’s conductivity distribution in 2-D or 3-D. This can be exploited to provide a distributed measurement of any variable that affects conductivity. The image reconstruction algorithm is key in realising high spatial resolution EIT images [9]. Algorithms vary by application, with open-source software available for testing on real data [10].

The major challenge facing concrete monitoring applications is that existing EITS interrogators tend to be expensive ($15k USD), plug-in bench systems which are optimised for chemical analyses and medical imaging. There is a clear need for low-power, low-cost and portable EITS interrogators optimised for wireless concrete structural health monitoring if this technology is to gain wider industry application. Low-power, portable devices for corrosion and electrochemical testing of concrete has been demonstrated and examples have been summarised by Segura et al. [11], including a wireless potentiostat [12]. Electrical *resistance* tomography (ERT) has been demonstrated for crack detection in concrete via an electrically conductive paint (although the interrogators used were not outlined) [13]. No examples of a low-cost, low-power, portable EITS interrogator for crack detection and location in concrete could be found in literature at the time of writing.

Recent work by the present authors has outlined the use of alkali-activated material (AAM) as a self-sensing non-structural concrete repair. We have demonstrated that changes in concrete and AAM repair material moisture [14], temperature [15] and strain [16] are encoded as a measurable shift in the bulk electrical impedance of discrete samples (i.e., no tomographic imaging was demonstrated).

In this paper, we present ‘ConcrEITS’: a new low-cost EITS interrogator which can provided continuous and distributed measurements of concrete specimens via electrically conductive coatings, such as AAM repairs. The system is capable of both single 4-probe impedance and 16-probe tomographic impedance measurements of AAM patches at various AC driving frequencies. Here, we benchmark ConcrEITS against a commercial 4-probe impedance analyser before validating its ability to detect and locate temperature changes and concrete cracking in small lab-scale specimens. ConcrEITS is in the proof-of-concept stage, and results are demonstrated at small scale. Future work will look to demonstrate ConcrEITS at full scale.

## 2. Materials and Methods

### 2.1. Self-Sensing Repair Coatings for Concrete

#### 2.1.1. Brief Theory

**Figure 1:**
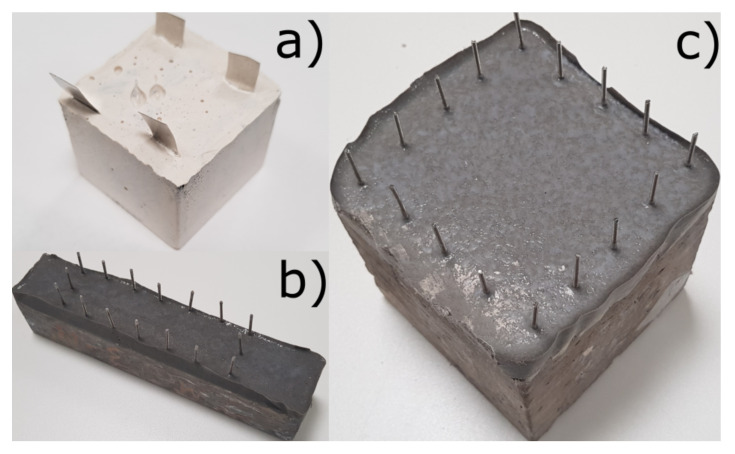
(**a**) Pure geopolymer sample used for benchmarking, (**b**) 40 × 40 × 200 mm beam and (**c**) 100 × 100 × 100 mm cube with instrumented AAM repair.

Alkali-activated materials (AAMs) are a class of curable cementitious materials produced by mixing an alkaline activator solution (such as sodium hydroxide and sodium silicate) with a precursor rich in silicon and aluminium (such as metakaolin). For more information on the fabrication of AAMs, their deployment and curing methods, and their use as repairs, see in [17,18]. Initially, AAMs are workable enough to pour but cure into a high strength brittle material, as shown in Figure 1a.

After curing, AAMs act as electrolytic conductors due to the presence of mobile sodium ions in their matrix [14]. Their inert electrical impedance, Z(ω,t), can be found by measuring the material’s voltage response, $V_{m}\left(\omega ,t\right)$ under an alternating current excitation $I_{a}\left(\omega ,t\right)$ as

(1) $$Z\left(\omega ,t\right)=\frac{V_{m}\left(\omega ,t\right)}{I_{a}\left(\omega ,t\right)}$$

where ω is the frequency term of the applied alternating current. The AAM’s impedance, Z(t), will vary due to measurands including temperature, humidity, strain, and sodium chloride [18]. Alternating current (AC), rather than direct current (DC), is used to prevent migration of the sodium ions through the matrix.

#### 2.1.2. Mix Design

The large number of variables involved in AAM mixing can cause contradicting conclusions from similar studies. Simple factors such as mixing technique and duration or even location of sourced alumnisilicate can change the mechanical properties of the final product [19,20]. It is likely that the “optimal” mechanical performing mix design will be unsuitable for certain applications. For these reasons, generally a trial and error approach to mix design (with reference to previous reports) for a particular application is required.

In this work, the focus is on acquiring a pourable and strong surface coating that can adhere well to a concrete specimen until cracking, with sufficient conductivity for sensing. The three main factors in a mix design are as follows:

- Solid:liquid ratio
- Na${}_{2}$SiO${}_{3}$:NaOH ratio
- Additives

Metakaolin was chosen over other alumnisilicates (fly-ash, Blast furnace slag (BFS)) for four reasons:

1. Requires a higher liquid content which increases conductivity [21].
2. Has more consistent particle sizes than fly-ash [22].
3. Is a natural resource compared to fly-ash (by-product of coal combustion) and BFS (by-product of iron/steel smelting).
4. More suitable as a repair than BFS [23].

However, Zhang et al. [24] suggest replacing a small quantity of metakaolin with fly-ash will improve compressive strength and reduces shrinkage. For this reason, 10% of metakaolin was replaced by fly ash. To make the metakaolin, kaolin clay sourced from the Southwest of England, UK was calcined at 800 °C for a duration of 2 h in an electric furnace. A solid:liquid ratio of 0.75 was chosen as this provided adequate workability for pouring but is high enough to minimize impact on compressive strength (0.8 is optimal [25]). A Na${}_{2}$SiO${}_{3}$:NaOH ratio of 2 was used as this should provide the best compressive strength, plus the 10 M concentration of Na${}_{2}$SiO${}_{3}$ provides sufficient conductivity [21,26]. For additives, sand at 50% of solid mass was added as this is known to improve both compressive strength and adhesion [27], with 50% being the optimal quantity [28]. Finally, polyvinyl alcohol (PVA) fibres at 0.5% of solid mass will both reduce shrinkage and improve adhesion to the substrate [29].

Every ingredient, their mass contribution and an example mass for the mix design used in this work is given in Table 1. For information on the composition of the alumni-silicates used, refer to our previous work: metakaolin [16], fly-ash [30].

Small 30 mm × 30 mm × 25 mm prism samples of this mix design were crushed in a compression machine. These experiments will provide a general indication of the compressive strength of the material. BS EN 12190:1999 requires 40 mm cubes to be tested, but this could not be done with the equipment available. Should this material be used in a real world application, a more extensive mechanical analysis should be performed. From a total of 13 prisms, an average compressive strength of 26 MPa was gained, with a standard deviation of 6 MPa. This meets BS EN 1504:1999 for non-structural repair of concrete (≥15 MPa).

#### 2.1.3. Application to Concrete

Prior to the AAM being applied, the concrete surface was wire brushed to improve adhesion. The AAM repairs were then applied manually within a temporary mould attached to the concrete surface. Stainless steel electrodes (plates or bolts) were inserted to allow for later electrical coupling to the impedance analyser/ConcrEITS instrument. Samples were then cured at room temperature in sealed containers for 28 days. This produced concrete prism and cube samples (Figure 1b,c) with adhered self-sensing repair patches. For testing, samples are wrapped in plastic film to maintain constant moisture level within the AAM.

### 2.2. ConcrEITS: Design and Benchmarks

#### 2.2.1. 4-Probe Impedance Measurement: Theory

Low-cost electrical impedance measuring devices for concrete have gained some interest in recent years: Corva et al. [32] developed a miniaturised resistance measuring device for chloride ingress measurement in concrete and Kaur et al. [33] developed a low cost electro-mechanical impedance device for damage detection in concrete using piezo-electric materials.

**Figure 2:**
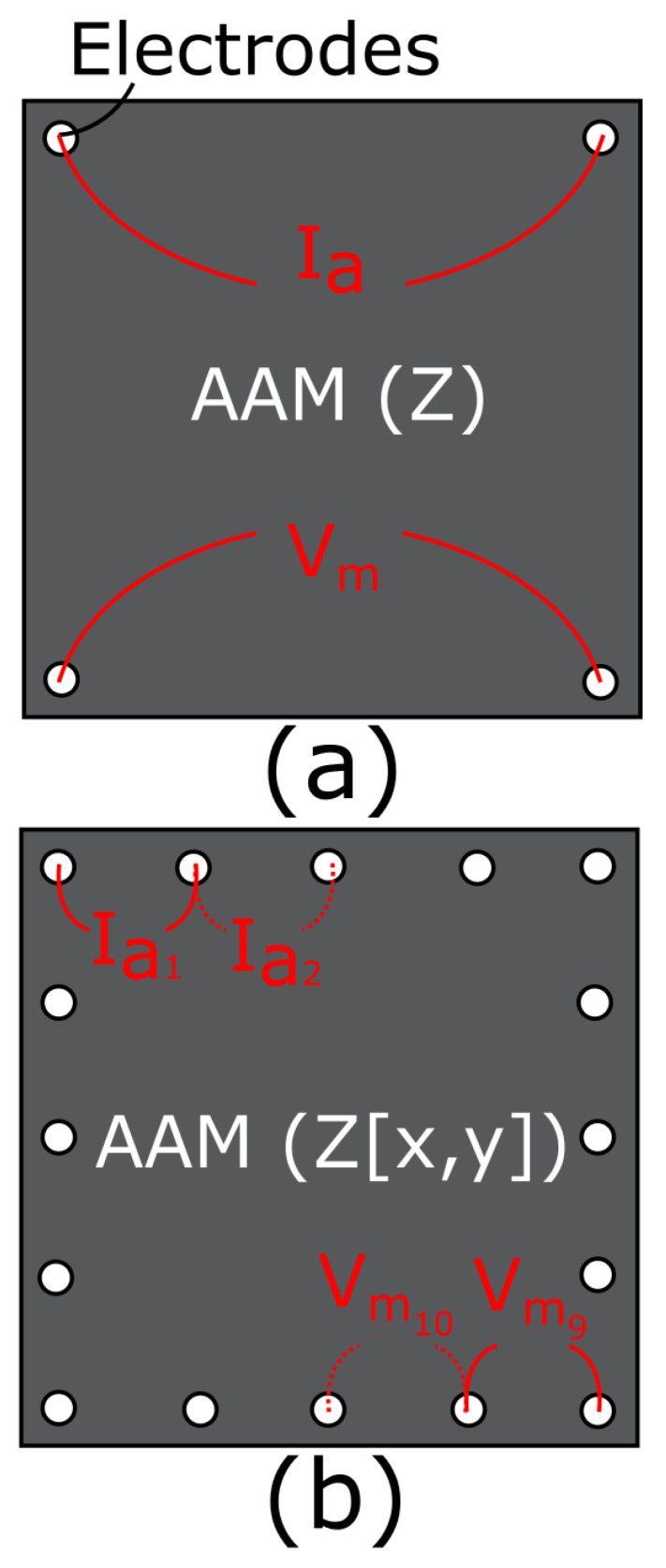
Configuration of electrodes in test samples: (**a**) VDP method for measuring impedance, *Z* of a single 4-probe cell, (**b**) serial tomographic measurement of 16-probe cell, $V_{a}$ is applied over a single electrode pair (1 shown), while $V_{m}$ is measured over all other 15 pairs (9 shown). This is repeated for every combination to give impedance array, Z[x,y] where *x* is applying pair and *y* is measuring pair.

The electrical impedance of a specimen can be monitored via 2-, 3-, or 4-probe AC excitation (or DC excitation if electrical resistance is the quantity of interest). For an extensive overview of these methods, along with examples of when they should be deployed, see [34]. In this work, focus will be on 4-probe AC excitation using the Van der Pauw (VDP) method [35], as illustrated in Figure 2a.

In its ideal case, the VDP method consists of small “point” electrodes placed exactly in the corners of a thin, cloverleaf shaped cell. However, these ideal conditions are rarely even approximately met in real applications: electrodes have a finite area, cannot be placed exactly in the corners, and AAM patches need to have a suitable shape and thickness to be able to function as a repair. Weiss [36] states that the resistivity of a cell can still be accurately measured without meeting these conditions, for a square/rectangular cell and electrode array.

Equation (1) can be used to assess the 4-probe impedance measurement illustrated in Figure 2a. An applied current $I_{a}\left(\omega ,t\right)$ results in a measured voltage $V_{m}\left(\omega ,t\right)$ over the two opposite electrodes. This results in a bulk average measured impedance for the entire patch.

For AAMs, the electrical impedance consists of a resistive ($Z_{R}$) and a capacitive ($Z_{C}$) element [14], and there is, therefore, a phase difference, ϕ=ωΔt, between current and voltage, and a magnitude difference given by the impedance modulus, $\left|Z\right|=\frac{|V_{m}|}{|I_{a}|}$.

#### 2.2.2. ConcrEITS Instrument Design

**Figure 3:**
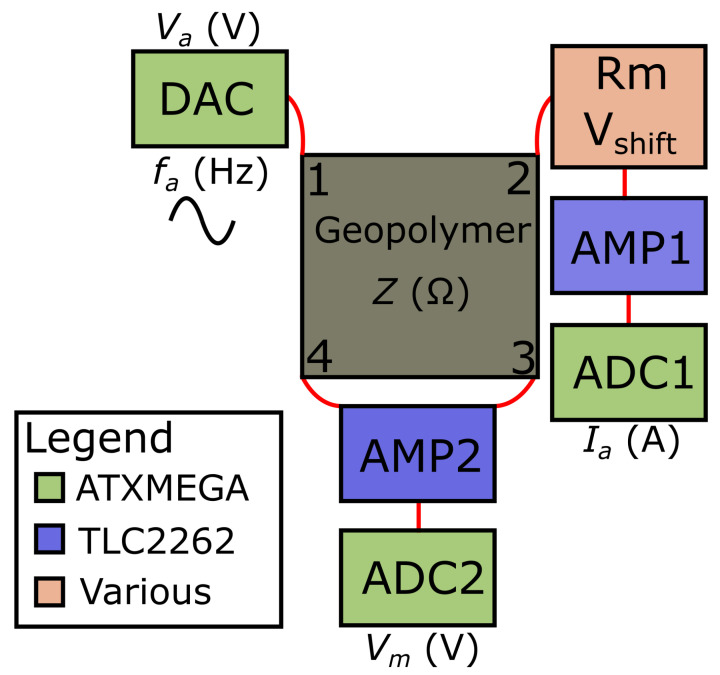
Simplified block diagram of ConcrEITS: a DAC from the MCU is used to apply an AC voltage, $V_{a}$, of varying frequency, $f_{a}$, which results in the applied current, $I_{a}$, over the excitation electrodes 1 and 2. A voltage is measured over opposing electrodes 3 and 4, $V_{m}$, to give the impedance of the AAM, *Z*. Voltage measurements are taken using the MCUs ADCs following suitable amplification by a TLC2262.

Version 1 of our circuit design was intended to interrogate discrete patch repairs via 4-probe electrical impedance spectroscopy (EIS). In EIS, impedance measurements are carried out over a range of excitation frequencies, ω. Interrogating concrete or a self-sensing repair’s response at various frequencies allows measured variables (e.g., moisture and sodium chloride contamination) to be elucidated and characterised if each measurand has a frequency-dependent response. To perform 4-probe EIS, a variable frequency voltage, $V_{a}$, is applied at the excitation electrodes and the resulting applied current is measured, $I_{a}$. At the two opposing electrodes, a summed voltage is also measured $V_{m}$. This is illustrated in Figure 3.

To carry out these tasks, and keep costs low, an ATXMEGA128A4U [37] micro-controller unit (MCU) was chosen. The on-board 12 bit digital-to-analogue converter (DAC) is used with a lookup table which is accessed after varying time periods to produce the required multi-frequency applied voltage sinusoid, $V_{a}\left(\omega ,t\right)$. The applied current, $I_{a}$, is found by running $V_{a}$ over a variable measurement resistor, $R_{m}$, before being amplified ($A_{1}$) using a TLC2262 chip and measured by an on-board 12-bit analogue-to-digital converter (ADC), $V_{ADC1}$. $I_{a}$ is calculated in postprocessing as

(2) $$I_{a}=\frac{\frac{V_{ADC1}}{A_{1}}}{R_{m}}$$

The measured voltage, $V_{m}$, is found via a summing amplifier ($A_{2}$) and then measured via a second ADC, $V_{ADC2}$. It can therefore be calculated, also in postprocessing, via $V_{m}=\frac{V_{ADC2}}{A_{2}}$.

The minimum conversion times of ≈7 μs and ≈3.5 μs for the DAC and ADC, respectively, mean that our maximum excitation frequency is $f_{a}=\frac{\omega }{2\pi }=$ 4.7 kHz for a 20-sample sinusoid.

Data logging is done via a serial interface UART-to-USB device, or using a UART-to-bluetooth module when wireless communication is required. The device is compatible with battery operation, as it is low power, drawing (≈95 mW), and uses a 3.4 V DC supply.

The total combined cost of components and manufacturing of a single ConcrEITS PCB is $50 USD at low fabrication volumes.

#### 2.2.3. ConcrEITS: Benchmarking

ConcrEITS was benchmarked against a commercial interrogator (which costs $16k USD). Benchmarking was conducted on pure AAM block samples, as shown in Figure 1a). These samples are small (30 × 30 × 25 mm) to keep the impedance relatively low and distance between electrodes short. This will allow accurate measurements from both interrogators. Should larger samples be used then benchmarking should be repeated for larger samples to ensure measurement accuracy remains the same. The impedance of these samples should be similar to those used for crack detection experiments. Benchmarking ConcrEITS using a single 4-probe impedance measurement instead of tomographic impedance measurements should be sufficient as tomography is simply the act of taking these 4-probe measurements over various electrode configurations. Furthermore, no all-in-one tomography impedance interrogator is widely available commercially at the time of writing. A separate multiplexer would be required in order to carry out tomography using the commercial interrogator, adding to the cost. Both devices were programmed to perform EIS sweeps from $f_{a}$ 100–1200 Hz. This range should be sufficient enough to vary the samples impedance and is within ConcrEITS capabilities.

**Figure 4:**
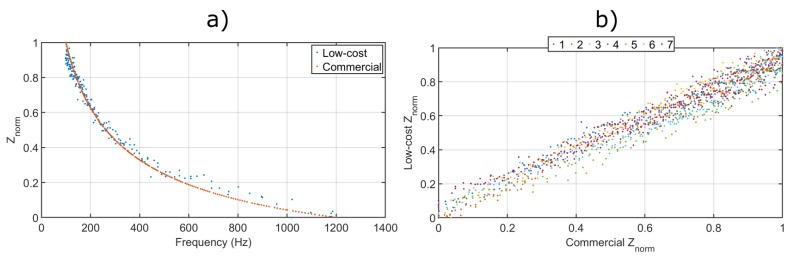
Results from interrogator comparison, (**a**) normalised impedance magnitudes from a single sample EIS, and (**b**) the impedance magnitudes measured at all frequencies for seven individual samples.

Results are shown in Figure 4, where all impedance magnitude values have been normalised as

(3) $$Z_{norm}=\frac{\left|Z(f_{a})\right|}{\left|Z(100\;Hz)\right|}$$

Figure 4a shows an example response obtained by both interrogators for a single sample. It is clear that the commercial interrogator is more accurate and precise than the low-cost ConcrEITS. However, ConcrEITS is able to follow the trend of impedance changes fairly well and would provide an acceptable estimate of a sample’s impedance.

For a closer comparison, Figure 4b shows the impedance responses measured over seven individual AAM samples. The root mean square error (RMSE) between low-cost (ConcrEITS) and the commercial interrogator is ±5.4%, which is adequate for our application.

### 2.3. ConcrEITS: Implementing Tomography

With the successful demonstration of ConcrEITS for 4-probe EIS, the next stage was to look to the requirements for distributed impedance mapping via electrical impedance tomography (EIT).

#### 2.3.1. Electrical Impedance Tomography: Theory

EIT can be considered as the implementation of the impedance measurement outlined in Section 2.2.1, but over a larger number of electrodes placed around the perimeter of the sample. This is usually done via multiplexing, and is illustrated in Figure 2b for 16 electrodes. Every combination of electrodes is serially measured to provide a matrix of impedance values Z(x,y), where *x* is the applying pair of electrodes, and *y* is the measuring pair. EITS is an extension of EIT, in which the frequencies are also altered during the measurement.

Historically, when discussing EIT, the electrical conductivity distribution, σ is described. This quantity is inversely proportional to the magnitude of the electrical impedance, |Z|, described above.

The objective of difference imaging in EIT is to produce a reconstructed colour contour image to analyse the change in conductivity (or impedance) distributions over time, relative to some baseline measurement. In contrast to absolute imaging, difference imaging is not sensitive to errors, such as electrode misplacements, misshapen bodies, and inhomogeneous materials. All of which are common in practical civil engineering applications. These benefits compensate somewhat for the reduced level of accuracy and spatial resolution compared to absolute imaging.

An in depth discussion of impedance mapping is provided in [38]. Briefly, EIT can be split into two main parts: (1) the forward problem and (2) the inverse problem. These are summarised below.

##### Forward Problem

The forward problem is the prediction of the boundary voltages of a body based on its modelled conductivity distribution. It can be stated in simplified terms as

(4) V=F(σ)+n

where *V* is a vector of observed electrode potentials, F(·) is a function which maps conductivity, σ to those electrode potentials, *V*, and *n* is some measurement noise.

At a deeper level, the equation which is being solved is the nonlinear Laplace equation:

(5) ∇·(σ∇μ)=0 (within the body,Ω)

where σ=σ(a) is the conductivity distribution over the body, and μ=μ(a) is the voltage potential at any location, *a* in the body, Ω.

To gain unique solutions to Equation (5), boundary conditions are set: that is information about the electrodes which are being used to interrogate the body. The model chosen for the boundary conditions is the complete electrode model (CEM), which describes among other parameters, electrode contact impedances, and the voltages and currents between multiple electrodes.

The forward problem is then to solve the CEM with Equation (5) over the area of the body. In practice, this is often achieved via discretisation of the body using the finite element method. The body is usually meshed into triangular elements. This converts the mathematically complex forward problem into a series of equations to be solved by a computer.

The maturity of EIT in the medical field means that there are already available methods to solve the forward problem using open-source software. One example is the Electrical Impedance Tomography and Diffuse Optical Tomography Reconstruction Software (or EIDORS), which is used in this work.

##### Inverse Problem

The purpose of the inverse problem is to utilise the forward model to estimate the conductivity change, δσ, following an event: in this paper, that could be a change in temperature of the AAM repair, or a crack. If we define an initial baseline conductivity measurement as $\sigma (t=t_{0})$, and a second conductivity measurement after time $t_{1}$ as $\sigma (t=t_{1})$, then the change is

(6) $$\delta \sigma =\sigma \left(t_{1}\right)-\sigma \left(t_{0}\right)$$

In the difference imaging method, a linearisation model is used to approximate the difference using Equation (4) as

(7) δV=Jδσ+δn

where δV = $V\left(t_{1}\right)-V\left(t_{0}\right)$ is the difference in voltage measurements, δn is any change in measurement noise, and *J* is the Jacobian matrix of mapped conductivities to voltages from the forward model. From this, the objective is now to find δσ, which is done via minimisation:

(8) $$\delta \sigma_{min}=\underset{\delta \sigma }{arg min}[||J\delta \sigma -{\delta V||}_{2}^{2}+p_{\sigma }\left(\sigma \right))]$$

where $\delta \sigma_{min}$ is the minimised change in conductivity and $p_{\sigma }\left(\sigma \right)$ is a regularisation term.

The inverse problem is ill-posed, because it has non-unique solutions. In other words, multiple conductivity distributions could lead to identical boundary voltages. The regularisation term $p_{\sigma }\left(\sigma \right)$ is therefore required to obtain stable solutions. Tikhonov regularisation is the most common method, and incorporates the assumption that the conductivity distribution is smooth [39]. Tikhonov regularisation was used for simplicity, and using other methods could improve results in future.

#### 2.3.2. ConcrEITS: Multiplexing Hardware Implementation

**Figure 5:**
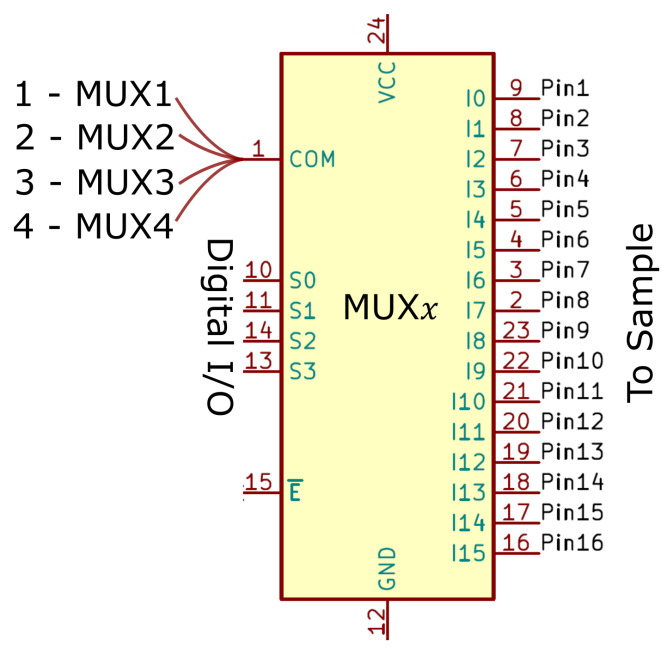
Multiplexer wiring diagram for ConcrEITS. Four multiplexers are used to switch the 4-probe signals over any combination of the 16 electrodes, as in Figure 2b. Each multiplexer is switched using 4 digital outputs from the MCU.

**Figure 6:**
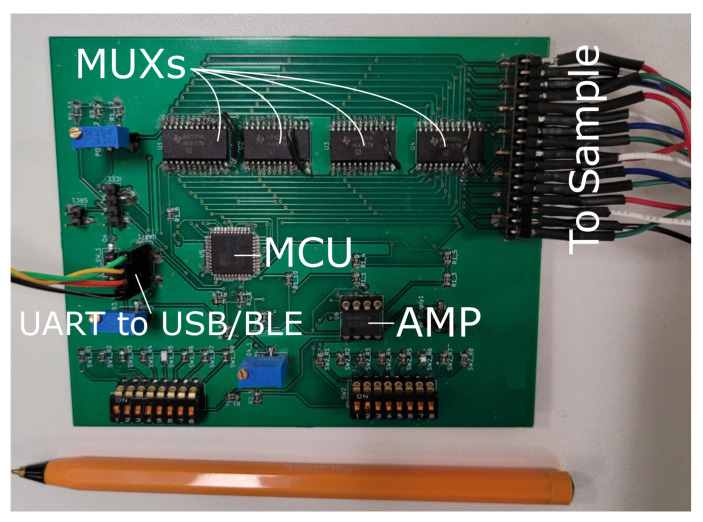
PCB of final ConcrEITS circuit with addressing wires. A UART to USB or UART to BLE module is used for data transmission.

The only additional hardware requirement to realise tomographic imaging is to perform impedance measurements over multiple pins via multiplexing. Due to the small size of the samples in this work, 16 pins were assumed to provide enough spatial resolution without overcomplicating the circuit. For larger AAM repairs samples, more electrodes may be required. From Figure 3, each of the four connections (labelled 1-4) are interconnected to separate 16-by-1 multiplexers. Each multiplexer is controlled via four digital outputs from the micro-controller. This means each connection from the interrogator to the AAM repair can be switched to any of the 16 electrodes at any time. The full final circuit diagram is shown in Figure 5 and the PCB is shown in Figure 6.

In the results presented in this paper, the firmware was updated to serially measure every combination of electrodes as previously illustrated in Figure 2b using a single frequency AC excitation of 1 kHz. A swept excitation can also be implemented. The electrode potentials, $|V_{m}|$ and injected currents, $|I_{a}|$, are extracted and fed into the EIDORS software, along with patch and electrode parameters. The total cost of ConcrEITS including multiplexing is around $60 USD at low production volumes.

## 3. Results

The output of EIDORS consists of a reconstructed contour image of the conductivity change between two states separated by time. For convenience, contour images are normalised and set to the same magnitude limits, allowing direct comparison between samples. Results are described in reference to change in conductivity, δσ. Given $\left|Z\right|\propto \sigma^{-1}$, a lower conductivity corresponds to a higher impedance. Low conductivities are represented as “black” in the contour and high conductivities as “white”.

### 3.1. Thermal Variations

Initial testing of ConcrEITS for EIT involved detection and location of temperature changes over a 100 mm × 100 mm patch. This test was chosen as it is non-destructive and could be repeated indefinitely. Our previous work [15] has shown that increases in AAM temperature cause increases in conductivity.

Results from a representative test are shown in Table 2. Heat is applied to the surface of the patch using heated ceramic dishes. In test T1, the dish is placed in the top right of the patch. The heat is removed after a period of time and the patch is allowed to cool before placing heat in a different location for subsequent tests T2 and T3.

The tomographic reconstruction shows that the resulting thermal variations are detectable from the electrical measurements. However, it is clear that discretely located temperature changes are dispersed, due to the low thermal conductivity of the AAM and the high thermal inertia of the concrete substrate. This may be why the system is unable to distinguish between two heated dishes during test T3.

Although interesting, these results show that despite being reversible, temperature mapping has its limitations as a testing and validation procedure for this system. In future, if temperature becomes the intended measurand, a thermocouple will be embedded within the AAM during testing to better understand heat dispersion in the AAM.

### 3.2. Crack Detection and Location

**Figure 7:**
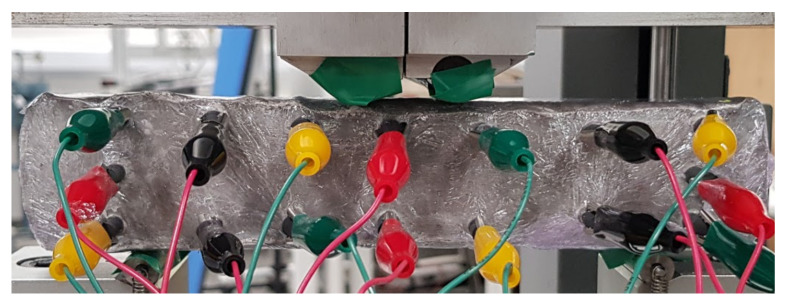
Small-scale beam bending experimental setup, with 16 electrodes interrogating an AAM patch on the concrete surface. Green tape was used to insulate the loading machine from the sample.

To test damage detection and location using ConcrEITS, small-scale concrete beam bending experiments were carried out, as shown in Figure 7. Loading machine contacts were insulated from the sample. AAM was applied to six 40 mm × 40 mm × 200 mm singly reinforced concrete beams, producing samples denoted SB1, SB2,..., SB6. Beams underwent linear 4-point bending (displacement rate = −0.01 mm/s) until cracks were visually distinguishable. Tomographic measurements of the system were taken continuously throughout.

**Figure 8:**
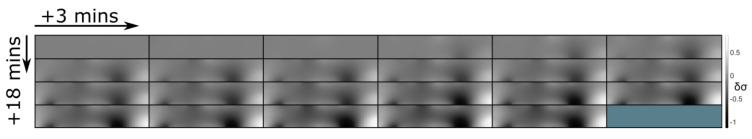
Sample SB5 tomographic reconstruction over time: each rectangle in the grid represents a single tomographic impedance difference image against the initial state. Each image frame represents the passing of 3 min in time.

Figure 8 shows sample SB5 image reconstruction over time. The system is clearly able to detect and locate cracks as they appear and begin to widen as load is increased over time. As expected, cracking leads to a localised decrease in conductivity (or increase in impedance), as it disrupts the pathways for ions migration in the AAM and concrete.

Table 3 shows a final photograph of the AAM surface of each sample (SB1 to SB6) after testing is complete, and compares it with the final tomographic image reconstruction. Electrodes 1 through 16 are numbered. In every case tested, cracks are detected and located successfully at their initiation and exit points, i.e., where the cracks propagate between electrodes.

For example, sample SB1 cracked between electrodes 11 and 12 which then propagated upwards to between electrodes 4 and 5. This is correctly measured from the tomographic reconstruction image. In cases where multiple cracks occur, such as SB5 which cracks between electrodes 10 and 11, 14 and 15, then 3 and 4: these are all observed in the tomographic reconstruction, with black contours appearing in these locations representing a decrease in conductivity. However, it is clear that accurate quantification of crack shapes and widths cannot be ascertained from these reconstructions.

We believe there are two main reasons for this. First, difference imaging is inherently less accurate than absolute imaging [8], therefore; we would need to minimise the associated errors (electrode misplacements, misshapen body, inhomogeneity) and model the electrode contact impedance to gain more accurate representations of the cracks through absolute imaging. Second, accuracy of the overall system itself, including interrogator voltage measurements and number of electrodes, will affect the resolution of the image reconstruction.

Nevertheless, ConcrEITS has demonstrated that is able to generally detect and locate cracks, and provide some measure of severity. Furthermore, the system provided no spurious results; there were no false positives or negatives. That is, no cracks were undetected and there were no large decreases in conductivity at locations without cracks. Overall, these results show that the low-cost, low-power ConcrEITS shows promise as a crack detection system for AAM repairs and potentially other self-sensing coatings which encode measurands as a conductivity change.

## 4. Discussion

Results in this paper represent a validation of ConcrEITS as a low-cost distributed crack detection system using AAM self-sensing repairs. This section will first analyse the results before discussing various parts of the research and how they could be improved for the future.

### 4.1. Analysing Results

Initial testing attempted to measure distributed temperature changes in a 100 mm × 100 mm patch. This sought a simple, fast, and repeatable experiment to validate the interrogator measurements. However, it is clear from the thermal variation images (Table 2 that distributed temperature changes are not detectable using ConcrEITS. A general change in temperature can be measured, but location is indistinguishable.

To validate crack detection, various small scale concrete samples (40 mm × 40 mm × 200 mm) beams with manually deployed AAM patches over a single surface were tested. Results show that distributed crack measurements over time are possible with ConcrEITS, although locations are general and exact crack widths, shapes and lengths are indistinguishable. Nevertheless, these results show promise for the use of ConcrEITS and AAM on large area concrete as a distributed crack detection system.

### 4.2. Future Work

#### 4.2.1. Improve Consistency of AAM Deployment

From Figure 1b,c it is clear the deployment of AAM is inconsistent: electrodes are misaligned and edges are uneven. In future this will be improved by using custom made moulds and templates. Additionally, ongoing research into automated deployment may solve these issues [16].

#### 4.2.2. Up-Scaled Experiments

Research in this paper demonstrates the capability of ConcrEITS to detect and locate cracks in small scale samples. Identifying the location of a crack in such a small area has little to no use in an industrial context. The final objective of ConcrEITS is to perform distributed crack detection over a large area covered with an AAM. Future work will look to upscale the research in this paper to larger areas, working towards a site deployment. This will also require benchmarking against the commercial interrogator for larger samples.

#### 4.2.3. Increase Frequency of AC Excitation

In Section 2.2, ConcrEITS was compared to a commercial interrogator in 4-probe EIS over a small frequency range (100–1200 Hz). The max applied frequency of the commercial interrogator is 1 MHz, compared to ConcrEITS’ 4.7 kHz for a 20 sample sinusoid. The major limitation on this is the conversion time of the DAC (7 μs) and ADC (3.5 μs). For EIS measurements, the ability to apply much higher frequency signal may be required. It is proposed that in future work, a separate excitation circuit will be constructed to apply the AC voltage, removing the delay caused by the DAC. Using just the ADC for sampling, and taking less samples per sinusoidal period should allow use of an AC frequency of up to 100 kHz.

#### 4.2.4. Improve Measurement Accuracy

In terms of resolution, the MCU uses 12-bit ADC and 12-bit DAC, which for a $V_{cc}$ of 3.3 V gives ± 0.8 mV. For an excitation voltage magnitude of 0.1 V this is sufficient. However, the benchmark against a commercial interrogator resulted in a ±5.4% RMSE. In future, this error will be minimised. Implementation of filters such as high-pass filters and unity gain amplifiers may remove any background noise and improve the accuracy.

## 5. Conclusions

In this paper, we have presented ConcrEITS: a low-cost ($60 USD) interrogator for tomographic and spectroscopic impedance mapping of concretes coated with conductive coatings. The system was demonstrated for an alkali-activated material-based repair. ConcrEITS was able to measure impedance to ±5.4% RMSE when benchmarked against a commercial impedance analyser. It was able to map concrete temperature variations with some degree of success, although temperature measurements were blurred due to the high thermal inertia of the concrete substrate. The system was also able to detect and broadly locate cracking in small-scale concrete beams with no spurious results. Future work will look to further test the system with scaled up 1 m long concrete beams instrumented with larger patches.

## Figures and Tables

**Table 1:** Mix design for the self-sensing AAM repair material.

Material	Wt%	Example (g)
Metakaolin ${}^{1}$	32.02	273
Fly ash ${}^{2}$	3.17	27
Sand ${}^{3}$ (≤800 μm)	17.58	150
PVA fibres (3 mm Length) ${}^{4}$	0.35	3
Na${}_{2}$SiO${}_{3}$ solution ${}^{3}$	31.18	266
NaOH solution (10 M) ${}^{3}$	15.70	134

^1^ Kaolin sourced from CEMEX, London, UK calcined in-house, ^2^ CEMEX, London, UK, ^3^ Commercial material sourced in UK, ^4^ Engineering Fibre Co. [31], Changzhou, China.

**Table 2:** Thermal variance detection and localisation results from tomographic reconstruction.

Ref.	Heat Location Image	Tomographic Reconstruction
T1	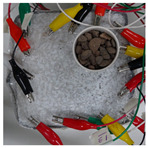	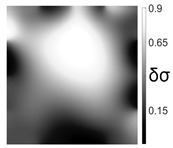
T2	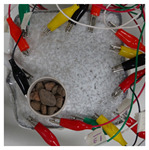	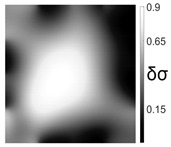
T3	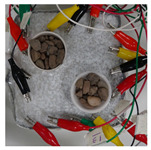	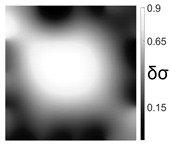

**Table 3:** Beam sample crack detection and localisation results from tomographic reconstruction.

Sample	Crack Image	Tomographic Reconstruction
SB1	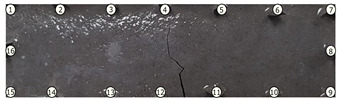	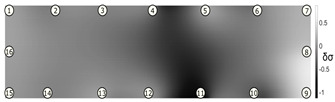
SB2	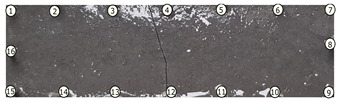	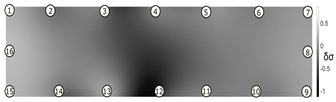
SB3	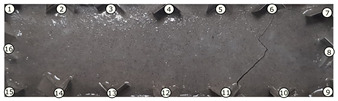	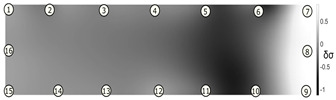
SB4	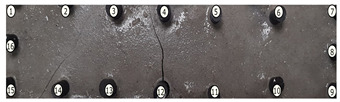	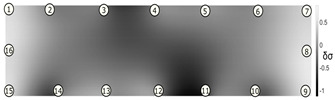
SB5	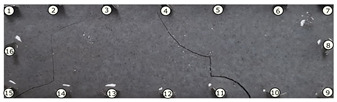	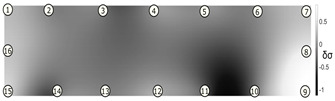
SB6	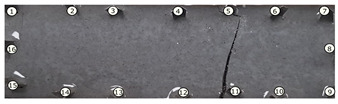	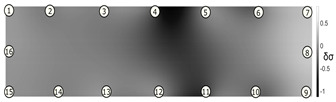

## Data Availability

Data sharing not applicable.

## Abbreviations

The following abbreviations are used in this manuscript:

EITS	Electrical impedance tomography and spectroscopy
AAM	Alkali-activated material
ERT	Electrical resistance tomography
EIS	Electrical impedance spectroscopy
EIT	Electrical impedance tomography
USD	United states dollar
VDP	Van der Pauw
MCU	Micro-controller Unit
ADC	Analogue-to-digital converter
DAC	Digital-to-analogue converter
UART	Universal asynchronous receiver-transmitter
USB	Universal Serial Bus
RMSE	Root mean square error
CEM	Complete electrode model
EIDORS	Electrical impedance tomography and diffuse optical tomography reconstruction
PCB	Printed circuit board
